# Supplementary material for: N-Alkylisatin-Loaded Liposomes Target the Urokinase Plasminogen Activator System in Breast Cancer
Source: Pharmaceutics. 2020 Jul 7;12(7):641. doi: 10.3390/pharmaceutics12070641 (PMC7408009; doi:10.3390/pharmaceutics12070641)
Supplement: Supplementary file 1 [file pharmaceutics-12-00641-s001.pdf]

pharmaceutics

Supplementary Materials for N-Alkylisatin-Loaded Liposomes Target the Urokinase Plasminogen Activator System in Breast Cancer

Lisa Belfiore, Darren N. Saunders, Marie Ranson and Kara L. Vine

Cell Surface uPA and uPAR Expression

Cells (1 x 10^5) in 100 µL PBS (pH 7.4, with 1% w/v BSA) were incubated with mouse anti-human uPA monoclonal antibody (ADI #394; Alpha Diagnostic International, TX, USA) at 10 µg/mL, mouse anti-human uPAR monoclonal antibody (DAKO #7294; Agilent Technologies, CA, USA) at 10 µg/mL or mouse IgG_1 monoclonal antibody (isotype control) (Merck #MABC002; Merck, Germany) at 10 µg/mL, for 45 min on ice, followed by three washes with ice-cold PBS (with 1% w/v BSA) and centrifugation at 300 x g for 5 min, after each wash. Cells were then incubated with donkey anti-mouse IgG-Alexa Fluor 488 polyclonal antibody at 2 µg/mL for 45 min on ice, in the dark, followed by three washes with ice-cold PBS (with 1% w/v BSA), as above. Cells were resuspended in 100 µL PBS and the fluorescence intensity of the Alexa-488-conjugated antibody was analyzed through flow cytometry (LSR II flow cytometer; BD Biosciences, CA, USA) (excitation 488 nm, emission collected with 515/20 band-pass filter). FlowJo software (version 10; Tree Star Inc., OR, USA) was used to evaluate cell-surface expression of uPA and uPAR. Analysis was completed by comparison to the IgG isotype control, to account for non-specific antibody binding to cells.

D Monolayer Cytotoxicity Assays

MCF-7 or MDA-MB-231 cells were seeded at a density of 5000 cells per well into sterile 96-well flat-bottom plates and incubated at 37 °C for 24 h, in an IncuCyte Zoom automated imaging instrument (Essen BioScience, MI, USA). Liposomes were incubated with cells for 72 h and the wells were imaged every 24 h by the automated IncuCyte imaging system. To determine cell viability at the experimental endpoint, CellTiter 96 Aqueous One Solution Cell Proliferation Assay MTS reagent (Promega Corporation, WI, USA) was added to each well, at a final concentration of 10% (v/v), incubated at 37 °C for 3 h and the absorbance at 490 nm was measured using a Spectramax spectrophotometer (Molecular Devices, CA, USA).

Pharmacokinetics and Biodistribution: Sample Preparation and Analysis

Liposome Preparation

Liposome thin films were prepared as described in the main manuscript and 400 µCi tritiated cholesteryl hexadecyl ether [cholesteryl-1,2-3H(N)] (3H-CHE) (PerkinElmer, MA, USA) (50 µCi per mL liposome) was added to the solution. The radioactivity of the preparations was measured using a scintillation counter (Tri-Carb 2810 TR Liquid Scintillation Counter; PerkinElmer, MA, USA) (10 µL liposome + 6 mL Ultima Gold). Prior to injection, the liposome preparations were sterile-filtered through a 0.22 µm pore PVDF filter and kept sterile for subsequent in vivo studies.

Tissue and Blood Preparation

Cohorts were euthanized at the designated time-points, post-treatment via CO_2 inhalation. Immediately after sacrifice, whole blood was collected by cardiac puncture, using 29-gauge insulin syringes and transferred into 1 mL EDTA animal collection tubes (Greiner, Austria). Blood was centrifuged at 500 x g for 15 min at RT, and 0.1 mL of plasma was transferred to a pre-weighed 7 mL glass scintillation vial. Kidneys, liver, spleen, lungs, and tumor, as well as the tail (to subtract activity remaining at injection site) were removed from each animal and transferred to individual pre-

weighed 20 mL glass scintillation vials. Vials were sealed and stored at 4 °C, until the tissues were processed. Solvable (PerkinElmer, MA, USA) was added to each vial to dissolve the tissues—0.4 mL for plasma; 2 mL for kidneys, spleen, lungs, and tail; 5 mL for liver; and 1 mL for tumors (where present). Vials were sealed and incubated at 60 °C for 1–3 h, with occasional agitation to dissolve tissues. Vials were then cooled to RT and hydrogen peroxide (30% v/v) was added to bleach the samples—0.2 mL for plasma, kidneys, spleen, lungs, and tail; 0.5 mL for liver; and 0.1 mL for tumor. Samples were allowed to stand for 30 min at RT to complete the reaction. Vials were then sealed tightly and incubated at 60 °C for 1 h. Vials were cooled to RT and 0.5 mL of each sample (0.25 mL for liver due to residual intense coloration) was transferred to a 7 mL glass scintillation vial. To this, 5 mL of Ultima Gold LSC (PerkinElmer, MA, USA) was added, vials were inverted to mix and the samples were temperature-adapted (25 °C) and dark-adapted for 1 h, prior to counting. Samples were analyzed using a Tri-Carb 2810 TR Liquid Scintillation Counter (PerkinElmer, MA, USA). Separate control vials for each tissue were prepared in parallel and contained the same volumes of each reagent, but in the absence of tissue. The raw counts for each sample were corrected for the amount of radioactivity remaining in the tail and are presented as the percentage of injected dose (ID) per gram of tissue (% ID/g) or percentage of ID in the whole tumor (% ID). Pharmacokinetic profiles were determined by plotting the % ID/mL remaining in the plasma over time, and fitted to a one-phase decay model using the GraphPad Prism V7 for Windows (GraphPad Software, CA, USA).

Absorption Spectra of *N*-AI and Liposome Phospholipid

The concentration of *N*-alkylisatin (*N*-AI) encapsulated in liposomes could not be determined by UV-Vis spectrophotometry and interpolation from a *N*-AI standard curve, as the liposome phospholipid interfered with the peak absorbance of *N*-AI at 310 nm and 435 nm.

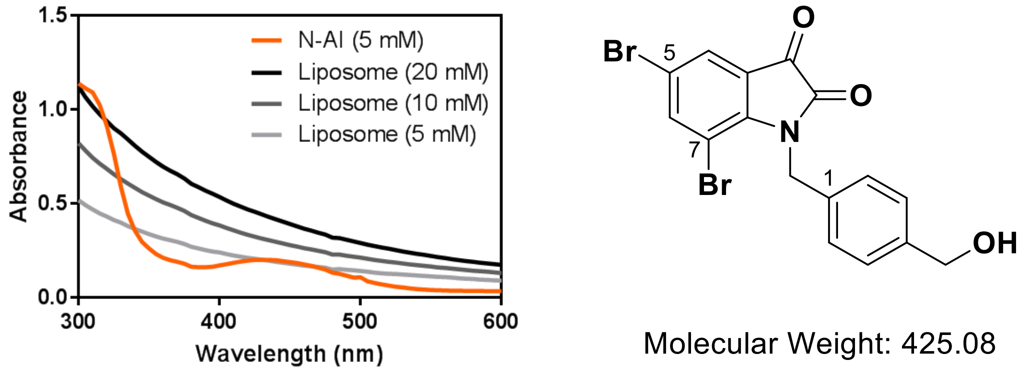

Figure S1. Liposome phospholipid interference with *N*-AI absorption spectrum and chemical structure of *N*-AI. The phospholipid of empty liposomes interferes at 310 nm and 435 nm, the two peak absorption wavelengths for *N*-AI.

HPLC Standard Curve for *N*-AI Quantification

The concentration of *N*-alkylisatin (*N*-AI) loaded into liposomes was determined by high performance liquid chromatography (HPLC), which showed an *N*-AI concentration of 2.2 mM, equating to 43.1% drug loading (% w/w), based on the starting amount of *N*-AI used in the liposome preparation. Concentration was determined by interpolation from an *N*-AI standard curve.

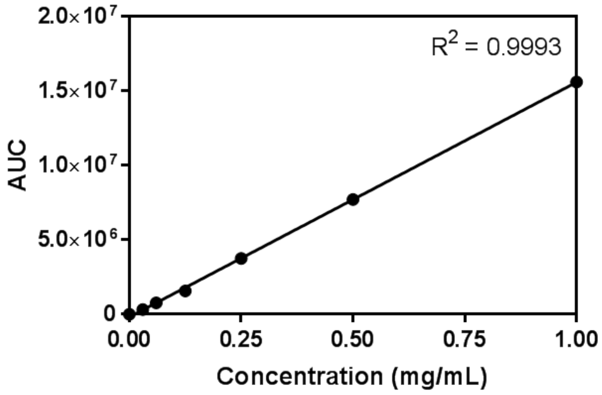

Figure S2. HPLC standard curve for quantifying *N*-AI encapsulated in liposomes.

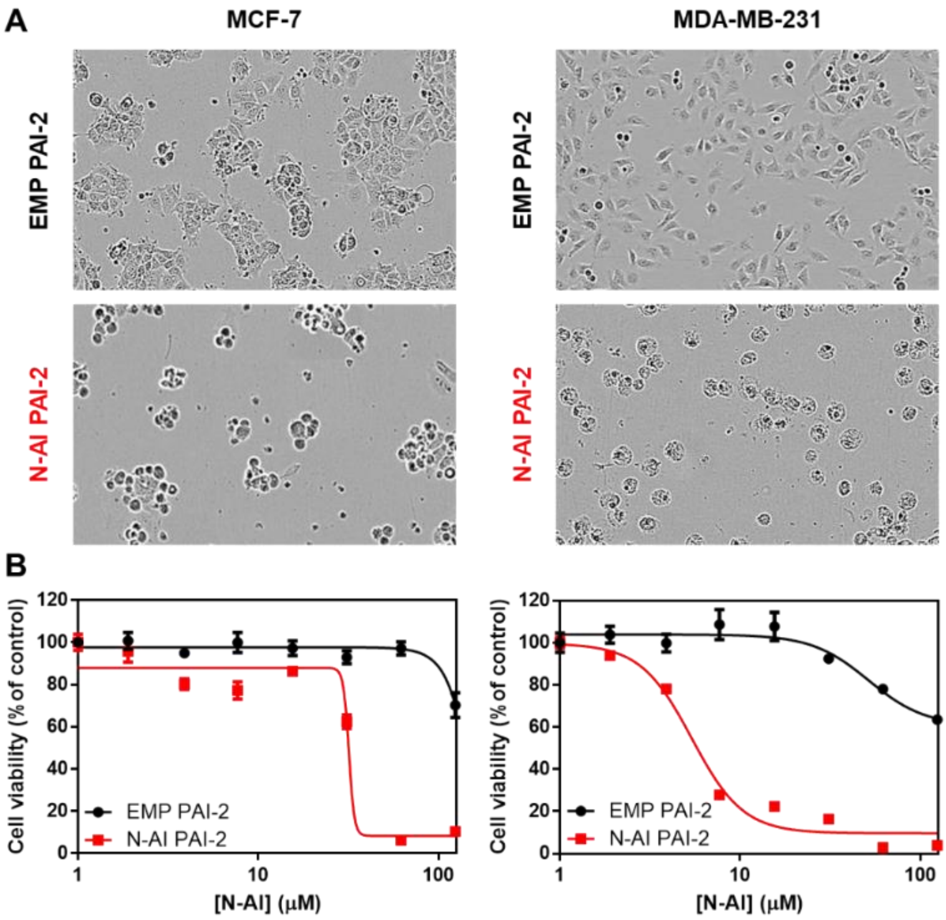

Figure S3. Cytotoxicity of N-AI PAI-2 liposomes. In vitro cytotoxicity testing of empty PAI-2 liposomes (EMP PAI-2) and N-AI-loaded PAI-2 liposomes (N-AI PAI-2) against MCF-7 (low uPAR) and MDA-MB-231 (high uPAR) breast cancer cell lines. (A) Representative images showing changes in cell growth and morphology, 72 h after treatment with liposomes at 62.0 μM N-AI, or equivalent phospholipid concentration (scale bars are 100 μm). (B) Dose-response cell viability curves via MTS assay at 72-h post-treatment. Data are the mean ± s.d. (n = 3).

Toxicology

Mice

Female BALB/c mice (Australian BioResources, Moss Vale) were housed in isolator cages at the University of Wollongong animal facility. Mice were given food and water ad libitum and kept on a 12-h light/dark cycle for the duration of the experiment. Mice were allowed to acclimatize for 2 weeks before the commencement of the experiment. All experiments were conducted in accordance with the 'NHMRC Australian Code for the Care and Use of Animals for Scientific Purposes', which requires 3R compliance (replacement, reduction, and refinement) at all stages of animal care and use, and the approval of the Animal Ethics Committee of the University of Wollongong (Australia), under protocol AE13/18.

Treatments

Liposome thin films were prepared as described in the main manuscript. Prior to injection, the treatment solutions were sterile-filtered through a 0.22 μm pore PVDF filter and kept sterile for subsequent in vivo studies. Mice were randomly allocated to treatment (free *N*-AI in DMSO vehicle, DMSO vehicle control, *N*-AI liposome, or EMP liposome control) groups (3 mice per cohort). Mice were injected with free *N*-AI at *N*-AI concentrations of 5, 8.5, 20, 25, and 40 mg/kg (or equivalent DMSO/PBS concentration; 16%-70%), and *N*-AI liposomes at *N*-AI concentrations of 1, 5, 10, 20, 50, and 100 mg/mg for *N*-AI liposomes (and equivalent EMP liposome concentrations). Treatments (100 μL) were administered intravenously via a single lateral tail-vein injection OR via multiple (5) lateral tail-vein injections over 5 days, to administer a total dose of 25 mg/kg free *N*-AI, and 50–100 mg/kg *N*-AI liposomes (and equivalent DMSO/EMP liposome controls). Mice that were deemed significantly (± 10%) smaller or larger in weight than their cage mates had their dose volume adjusted proportionally, based on their weight, relative to the average of their cage mates. Weight change was monitored over time for a total of 4 weeks. The maximum tolerated dose endpoints were defined as 15% loss of body weight (compared to the first day of treatment and sustained for >24 h), or clinical signs of morbidity (i.e., loss of appetite, activity or hunched posture, piloerection, and changes in gait). Graphs were generated using the GraphPad Prism V7 for Windows (GraphPad Software, CA, USA). Mice were euthanized at the end of the study by CO_2 overdose, and the major organs were harvested and weighed.

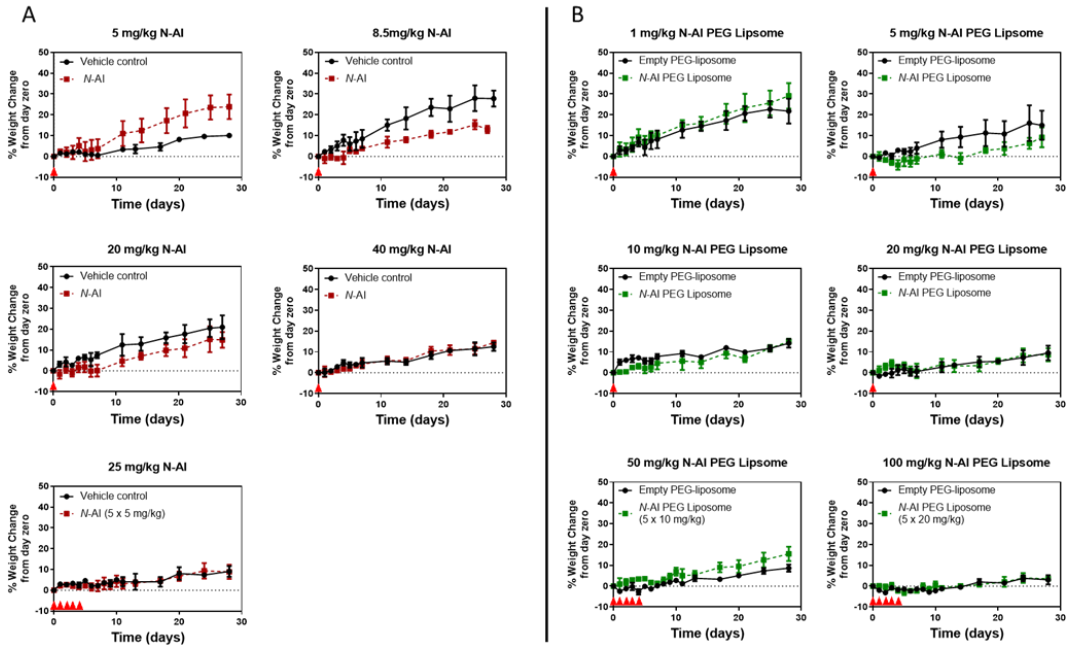

Figure S4. Toxicology testing of empty and *N*-AI-loaded liposomes in mice. Mice were treated with either a single bolus dose or multiple doses (indicated red by arrows) of (A) free *N*-AI or DMSO/PBS vehicle control, or (B) *N*-AI-loaded liposomes, or empty (EMP) liposomes at an equivalent

phospholipid concentration, and weight change was monitored over time. Data are the mean ± standard deviation ($n = 3$ per treatment cohort).

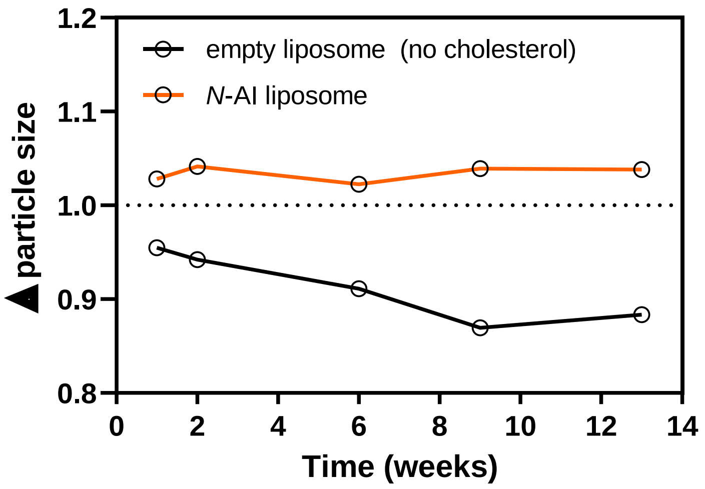

Figure S5. Stability of *N*-AI-loaded liposomes over time. The fold-change in particle size of *N*-AI liposomes and empty liposomes not containing cholesterol were determined using dynamic light scattering, over a period of 13 weeks.
